# Protections against the Risk of Airborne SARS-CoV-2 Infection

**DOI:** 10.1128/mSystems.00390-20

**Published:** 2020-06-16

**Authors:** Clement J. McDonald

**Affiliations:** University of California, San Diego

**Keywords:** COVID-19, HEPA filters, N95 respirator, route of infection, SARS-CoV-2, airborne microorganisms, negative-pressure rooms

## LETTER

Dietz at al. (1) call attention to issues that affect the spread of severe acute respiratory syndrome coronavirus 2 (SARS-CoV-2) within the built environment. Policymakers have emphasized the importance of contact transmission of SARS-CoV-2 strongly but airborne transmission minimally. The CDC recommends both contact and airborne precautions for CoV disease 2019 (COVID-19) patients (https://www.cms.gov/files/document/qso-20-09-all.pdf). The virus is present in the air of hospital rooms holding COVID-19 patients and in the hallways outside those rooms and exists in particles small enough to persist in the air (2). Further, airborne SARS-CoV-2 can remain infective for 3 hours (3). Body parts touched by air—the nose, pharynx, and lung—are the first to be infected, and those same parts supply the ambient air with large quantities of the virus when the infected patient coughs, sneezes, speaks, or breaths (4). SARS-CoV-2 does not have the multi-mile airborne reach of hoof-and-mouth disease (5). However, it does have a reach of 26 feet and a loft time of 3+ hours in simulation studies of coughs and sneezes (6). Policy makers should not discount the risk of airborne transmission.

Nominally, N95 respirators block 95% of particles larger than 3 μm. Coronaviruses are roughly 0.1 μm in size. Given the virus’s small size, Dietz et al. (1) and others have questioned whether N95 respirators block them. They do. N95 respirators exceed their nominal rating in NIOSH testing. They block particles as small as 0.1 μm with 98% efficiency (7) and viruses of 0.1 μm in direct-challenge tests (8).

The explosive spread of COVID-19 to cruise ship passengers mostly confined to their cabins and the fact that SARS-CoV-2 may loll in the room air of infected individuals raises the question of cabin-to-cabin transmission through ship ventilation systems (https://www.purdue.edu/newsroom/releases/2020/Q1/cruise-ship-ac-systems-could-promote-rapid-coronavirus-spread,-prof-says.html). Cruise ships recirculate shared air from many cabins without much filtering. One could ask a similar question about the rapid spread in some nursing homes. Virus testing of the ventilation ducts in nursing home hot zones may help to answer this question.

Dietz et al. (1) describe mechanisms to reduce the risk of airborne COVID-19 infection. HEPA filters have the same 3-μm pore size as N95 respirators but a greater, 99.7%, filtering efficiency. Installing them in the air handling systems of care systems will reduce the viral load in ambient air and eliminate the risk of air duct transmission mentioned above. Hospitals protect some spaces with HEPA filters. They should review their air system and add HEPA filters where needed around COVID-19 care areas.

Negative-pressure rooms continually exhaust room air to the outside. They shrink the room’s airborne viral load and keep infected air out of hallways and other rooms. Ideally, COVID-19 patients should all reside in negative-pressure spaces. However, in many cities, the number of such spaces will be inadequate (9). The Minnesota health department published a practical guide (https://www.health.state.mn.us/communities/ep/surge/infectious/airbornenegative.pdf) for making temporary negative-pressure rooms to fill this gap. Every hospital facility manager in COVID-19 hot zones should review the Minnesota guide.

We should employ all available defenses against airborne SARS-CoV-2.

## References

[1] Dietz L, Horve PF, Coil DA, Fretz M, Eisen JA, Van Den Wymelenberg K 2020 2019 novel coronavirus (COVID-19) pandemic: built environment considerations to reduce transmission. mSystems 5:e00245-20. doi:10.1128/mSystems.00245-20. 32265315 PMC7141890

[2] Santarpia JL, Rivera DN, Herrera V, Morwitzer MJ, Creager H, Santarpia GW, Crown KK, Brett-Major D, Schnaubelt E, Broadhurst MJ, Lawler JV 2020 Transmission potential of SARS-CoV-2 in viral shedding observed at the University of Nebraska Medical Center. medRxiv doi:10.1101/2020.03.23.20039446.

[3] van Doremalen N, Bushmaker T, Morris DH, Holbrook MG, Gamble A, Williamson BN, Tamin A, Harcourt JL, Thornburg NJ, Gerber SI, Lloyd-Smith JO, de Wit E, Munster VJ 2020 Aerosol and surface stability of SARS-CoV-2 as compared with SARS-CoV-1. N Engl J Med 382:1564–1567. doi:10.1056/NEJMc2004973. 32182409 PMC7121658

[4] Asadi S, Wexler AS, Cappa CD, Barreda S, Bouvier NM, Ristenpart WD 2019 Aerosol emission and superemission during human speech increase with voice loudness. Sci Rep 9:2348. doi:10.1038/s41598-019-38808-z. 30787335 PMC6382806

[5] Verreault D, Moineau S, Duchaine C 2008 Methods for sampling of airborne viruses. Microbiol Mol Biol Rev 72:413–444. doi:10.1128/MMBR.00002-08. 18772283 PMC2546863

[6] Bourouiba L 2020 Turbulent gas clouds and respiratory pathogen emissions: potential implications for reducing transmission of COVID-19. JAMA 323:1837–1838. doi:10.1001/JAMA.2020.4756. 32215590

[7] Rengasamy S, Shaffer R, Williams B, Smit S 2017 A comparison of facemask and respirator filtration test methods. J Occup Environ Hyg 14:92–103. doi:10.1080/15459624.2016.1225157. 27540979 PMC7157953

[8] Eninger RM, Honda T, Adhikari A, Heinonen-Tanski H, Reponen T, Grinshpun SA 2008 Filter performance of N99 and N95 facepiece respirators against viruses and ultrafine particles. Ann Occup Hyg 52:385–396. doi:10.1093/annhyg/men019. 18477653 PMC6768072

[9] Subhash SS, Baracco G, Miller SL, Eagan A, Radonovich LJ 2016 Estimation of needed isolation capacity for an airborne influenza pandemic. Health Secur 14:258–263. doi:10.1089/hs.2016.0015. 27447336

